# Posture Detection Based on Smart Cushion for Wheelchair Users

**DOI:** 10.3390/s17040719

**Published:** 2017-03-29

**Authors:** Congcong Ma, Wenfeng Li, Raffaele Gravina, Giancarlo Fortino

**Affiliations:** 1 School of Logistics Engineering, Wuhan University of Technology, Wuhan 430070, China; macc@whut.edu.cn; 2 Department of Informatics, Modeling, Electronics and Systems, University of Calabria, Rende 87036, Italy; rgravina@dimes.unical.it (R.G.); g.fortino@unical.it (G.F.)

**Keywords:** smart wheelchair, posture detection, smart cushion, pressure sensor, activity level assessment

## Abstract

The postures of wheelchair users can reveal their sitting habit, mood, and even predict health risks such as pressure ulcers or lower back pain. Mining the hidden information of the postures can reveal their wellness and general health conditions. In this paper, a cushion-based posture recognition system is used to process pressure sensor signals for the detection of user’s posture in the wheelchair. The proposed posture detection method is composed of three main steps: data level classification for posture detection, backward selection of sensor configuration, and recognition results compared with previous literature. Five supervised classification techniques—Decision Tree (J48), Support Vector Machines (SVM), Multilayer Perceptron (MLP), Naive Bayes, and k-Nearest Neighbor (k-NN)—are compared in terms of classification accuracy, precision, recall, and F-measure. Results indicate that the J48 classifier provides the highest accuracy compared to other techniques. The backward selection method was used to determine the best sensor deployment configuration of the wheelchair. Several kinds of pressure sensor deployments are compared and our new method of deployment is shown to better detect postures of the wheelchair users. Performance analysis also took into account the Body Mass Index (BMI), useful for evaluating the robustness of the method across individual physical differences. Results show that our proposed sensor deployment is effective, achieving 99.47% posture recognition accuracy. Our proposed method is very competitive for posture recognition and robust in comparison with other former research. Accurate posture detection represents a fundamental basic block to develop several applications, including fatigue estimation and activity level assessment.

## 1. Introduction

The continuous progress of ICT (Information and Communication Technology) is rapidly changing our daily life. Thanks to the high processing capabilities, reduced dimensions and low cost of modern microelectronics, a plethora of wearable devices are being realized to non-invasively support and monitor several human daily activities. Such devices play a central role in the so-called ubiquitous sensing [1], which aims at gathering data from the sensors and mining knowledge from them. Body Area Networks (BANs) can measure physical activity and biophysical signals [2] and pose the basis for a wide range of new user-centric applications in the domains of health care [3], remote monitoring [4], activity measurement [5], activity level assessment [6], user-centric wheelchairs [7], and health care service security [8].

In order to cope with the problems that elderly and mobility disabled individuals are facing, more effective care services, especially for wheelchair users, need to be provided. Several supportive devices have already been proposed to help such people keep an independent lifestyle. For this purpose, mobility-assistive devices, including smart wheelchairs, have recently begun to emerge. A smart wheelchair augments the functionalities of a traditional wheelchair by means of various technologies such as BANs [9], cloud computing [10,11], and multi sensor fusion [12].

Current research on smart wheelchairs is mainly focused on vital signals and physical activity monitoring [13], human–machine interface to control the wheelchair [14], obstacle detection and navigation [15], and wheelchair movement condition estimation [16].

This work is focused on a wheelchair assist system for mobility-impaired individuals that can recognize postures using a cushion based system. Pressure sensors are used to obtain data of the wheelchair user’s postures, several classification methods have been compared to determine an efficient classifier, and former sensor deployment methods have been compared against our proposed deployment to determine an optimal deployment method. In addition, some practical applications are discussed in the paper. If dangerous situations such as long-term abnormal posture are detected, the system can issue an alarm immediately; in addition, posture detection is a basic block towards activity level assessment.

The main contributions of this work are the following:

- An optimization method of pressure sensor deployment is proposed to more accurately detect sitting postures.
- An in-depth comparison of several classification techniques has been carried out to identify the best posture classifier based on pressure sensors’ smart cushion.
- In contrast with previous studies, the Body Mass Index (BMI) is among the considered parameters to evaluate the generality and robustness of the proposed deployment method across different body shapes.

The reminder of the paper is organized as follows. Section 2 discusses the related work on posture recognition using smart cushion; we describe and analyze the two main production technologies of smart cushions for pressure detection, the first is based on pressure sensor array and the second relies on the use of a few individual sensors deployed on the seat and backrest. Section 3 describes the architecture of the proposed system. Section 4 reports the system evaluation; data collection and experimental settings as well as optimal sensor selection methods are discussed here. Section 5 is about the results and performance analysis; we compared several classifiers and the results of our optimal sensor deployment are analyzed in comparison with the state-of-the-art. Finally, Section 6 concludes the paper and some future directions are anticipated.

## 2. Related Work

Most previous studies focused on monitoring physical activities of wheelchair users. Postolache et al. [17] proposed a system for monitoring activities performed on wheelchairs by analyzing inertial data. Hiremath et al. [18] developed a similar system but used gyroscopes for capturing wheelchair overturn and a wearable accelerometer to detect physical activity.

Interestingly, although wheelchair users spend most of their time sitting in the wheelchair, and it would be therefore important to analyze sitting postures and behaviors, fewer works have been devoted to these aspects. Wu et al. [19] proposed using ultrasound sensors to measure the distance between the user and wheelchair in order to recognize a number of sitting postures. Nakane et al. [20] focused on fatigue assessment based posturography and developed a pressure sensor sheet. Ding et al. [21] were interested in monitoring the status of the wheelchair including seat tilt, backrest recline, and seat elevation. In a previous work [22], we proposed a cloud-computing infrastructure based on the BodyCloud platform [23,24], and a pressure sensor cushion was used to monitor body postures of wheelchair users.

Due to its unobtrusiveness, several literature works adopted a pressure sensor-based cushion to recognize physical activity, posture and fatigue of individuals sitting on standard chairs or on wheelchairs [25]. Specifically, there are two main production technologies of smart cushions for pressure detection: the first one is based on a pressure sensor array, whereas the second one relies on the use of fewer individual sensors deployed on the seat and backrest.

### 2.1. Cushion with Pressure Sensor Array

Xu et al. [26] proposed a textile-based sensing system called Smart Cushion used for user’s sitting postures. Binary pressure distribution data was collected and the data model can be interpreted as binary representation of a gray scale image. Dynamic time warping has been applied to pressure distribution in order to recognize postures. There is also a representative sensor matrix called an I-Scan System developed by Tekscan [27]. The versatile I-Scan tactile pressure mapping system consists of over 2000 pressure sensors. It is a powerful tool that accurately measures and analyzes interface pressure between two surfaces. Tan et al. [28] used the commercially available pressure distribution sensor called a Body Pressure Measurement System (BPMS), also manufactured by Tekscan. Using principal components analysis to analyze a pressure distribution map as a gray scale image, sitting posture classification was successfully processed. Mota et al. [29] also used Tekscan; posture features were extracted using a mixture of four Gaussians and served as input to a three-layer feed-forward neural network. Nine different postures were recognized with 87.6% accuracy on average. Meyer et al. [30] presented their own capacitive textile pressure sensor array cushion to measure pressure distribution of the human body. They used a Naive Bayes classifier to identify 16 different sitting postures on the chair, with results similar to Tan. Multu et al. [31] proposed a robust low-cost non-intrusive seat; compared to the commercially-available Tekscan ConforMat system (over 2000 sensors) used in [28,30], they managed to reduce the number of sensors, and an optimal sensor deployment with 19 pressure sensors was adopted to recognize sitting postures. Kamiya et al. [32] used an 8×8 pressure sensor matrix inserted inside the foam filling of the chair to recognize sitting postures. Radial basis function and SVM algorithms have been used to recognize various postures. Xu et al. [33] presented the design and implementation of a low-cost smart cushion equipped with sensor array. Binary value of pressure distribution (i.e., when the pressure applied to a sensor is higher than a given threshold, its value is 1; otherwise, it is 0) was analyzed to recognize recognize nine postures in real time. Fard et al. [34] proposed a system for preventing pressure ulcers based on an 8×8 pressure sensor matrix for continuous monitoring of surface pressure of the seat.

All of the literature works analyzed, summarized in Table 1, detect and analyze pressure distribution on the cushion/mattress to recognize the postures. On the one hand, pressure sensor array technology allows for accurately measuring multiple pressure points on the seating surface; on the other, however, it leads to costly solutions.

### 2.2. Smart Cushions Based on Fewer Individual Pressure Sensors

Hu et al. [35] proposed PoSeat, a smart cushion equipped with an accelerometer and pressure sensors for chronic back pain prevention using a hybrid SVM classifier. Benocci et al. [36] proposed a method using five pressure sensors and k-Nearest Neighbour (kNN) was used to classify six different postures. Bao et al. [37] used a pressure cushion to recognize sitting postures by means of a density-based clustering method. Diego et al. [38] proposed a non-invasive system for monitoring pressure changes and tilts during daily use of the wheelchair. Pressure sensors have been deployed on the seat to detect pressure relief tilts and provide reminders to change posture to reduce the long-term risk of causing pressure ulcers. Min et al. [39] proposed a real-time sitting posture monitoring system based on measuring pressure distribution of the human body sitting on the chair. A decision tree is used to recognize five sitting postures; when a user deviates from the correct posture, an alarm function is activated. Zemp et al. [40] developed an instrumented chair with force and acceleration sensors to identify the user’s sitting posture by applying five different machine learning methods (Support Vector Machines, Multinomial Regression, Boosting, Neural Networks and Random Forest). Sixteen force sensor values and the backrest angle were used as the features for the classification, and the recognition results reached average accuracy of 90.9%. Barba et al. [41] used the postural changes of users to detect three kinds of affective states in an e-learning environment. Sixteen pressure sensors were placed on the chair, half on the seat and the other half on the backrest. Fu et al. [42] proposed a robust, low-cost, sensor based system that is capable of recognizing sitting postures and activities. Eight force sensing resistors (FSRs) were placed on chair backrests and seats, and a Hidden Markov Model approach was used to establish the activity model from sitting posture sequences. Kumar et al. [43] designed Care-Chair with just four pressure sensors on the chair backrest. The proposed system can classify 19 kinds of complex user sedentary activities, and it can also detect user functional activities and emotion based activities. Our former research [44] used three pressure sensors, two on the seat and one on the backrest to detect user’s postures in smart wheelchairs. We were able to recognize six different postures; results obtained with experimental evaluation showed high classification accuracy.

There are also two commercial smart cushions called DARMA [45] and SENSIMAT [46] that can monitor posture and sitting habits. Both of them are composed of six sensors and have similar deployment. DARMA is a general-purpose cushion equipped with fiber optic sensors that are much more sensitive and accurate than pressure sensors. It can track user’s posture and sitting time as well as heartbeat and respiration. Based on user’s particular behavior and habits, DARMA provides several functionalities such as actionable coaching, stand-up reminders, and posture advice. SENSIMAT is instead specifically designed for wheelchairs. The proprietary PressureRisk algorithm allows for monitoring sitting pressure and pushing data to the mobile device. It can send reminders to the user when it is time to move and can track movements and progress over time.

Each analyzed literature work, as shown in Table 2, proposes its own sensor deployment, achieving relevant results. However, to the best of our knowledge, a systematic study on the selection of the optimal sensor deployment on the cushion, and which classifier is most suitable for cushion-based posture recognition, is still lacking. In the following, we will introduce an optimal sensor deployment method to detect wheelchair user’s posture; in addition, we evaluate several classification techniques to identify the most effective ones for sitting posture recognition.

## 3. System Design

In the following, we describe our method for monitoring postures of smart wheelchair users. In particular, we designed a smart cushion to monitor sitting postures. The method serves as a basis for the development of a smart wheelchair system that can e.g., warn the user of long-term wrong/dangerous postures and, in case of emergency situations, alert relatives and caregivers to help the assisted user on time. In the following sections, we will describe the system in detail.

### 3.1. System Architecture

**Figure 1:**
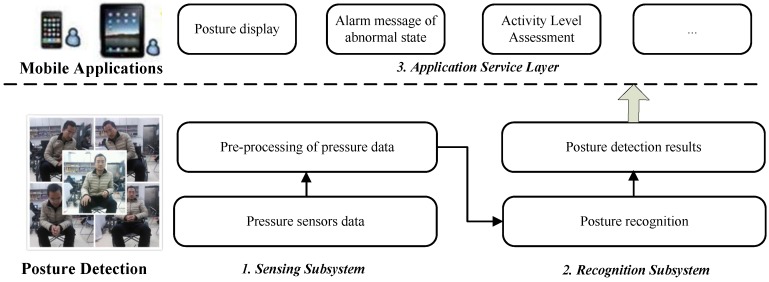
Architecture of the proposed system.

A layered architecture of the proposed posture detection system is depicted in Figure 1. The posture detection layer represents the core of the proposed system and it has been already implemented; several application services—outside the scope of this work—can be easily developed on top.

It is composed of two main layers:

- *Posture Detection Layer*: it includes two subsystems, (i) the *Sensing subsystem*, which uses pressure sensors deployed on the wheelchair to collect data generated by the weight of the body and (ii) the *Recognition subsystem* that, based on the Arduino platform (see Section 3.2), runs the posture recognition algorithm on the collected pressure sensor data. Posture detection results can therefore be fed to the application service layer.
- *Application Service Layer*: several (mobile and cloud-based) applications can be developed on top of the posture recognition subsystem and exploit the geo-location from dedicated mobile device services (such as GPS, WiFi, or cellular tower signal strength) to locate the user. Applications can display the sensing results, perform activity level assessment, alert the user of dangerous postures and, if necessary, send his/her geo-location and make emergency automatic voice calls to caregivers.

### 3.2. Hardware Design of the Smart Cushion

The smart cushion is composed of three main modules:

- *data sensing* (cushion equipped with pressure sensors);
- *data processing* (Arduino-based unit);
- *data transmission* (Bluetooth shield for Arduino).

**Figure 2:**
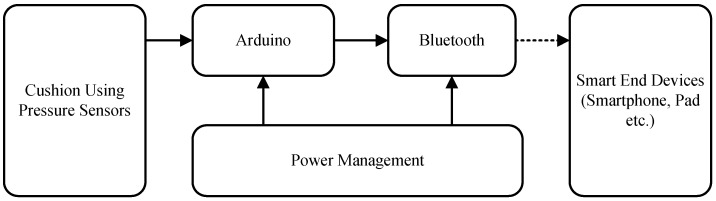
Hardware design of the smart cushion.

The hardware design of the smart cushion is shown in Figure 2.

The data sensing module is based on Force Sensitive Resistor pressure sensors (Interlink FSR-406) [47]. The resistance value is inversely proportional to the pressure applied on the sensing patch. Such sensors can be easily embedded into the chair textile or foam filling. We chose Arduino DUE [48] to construct the data processing module for its low energy consumption, high sampling rate and processing capabilities. After sensor data collection and processing, results are sent to the smartphone in wireless, using an Arduino Bluetooth HC-06 shield [49] that is attached to the processing module.

**Figure 3:**
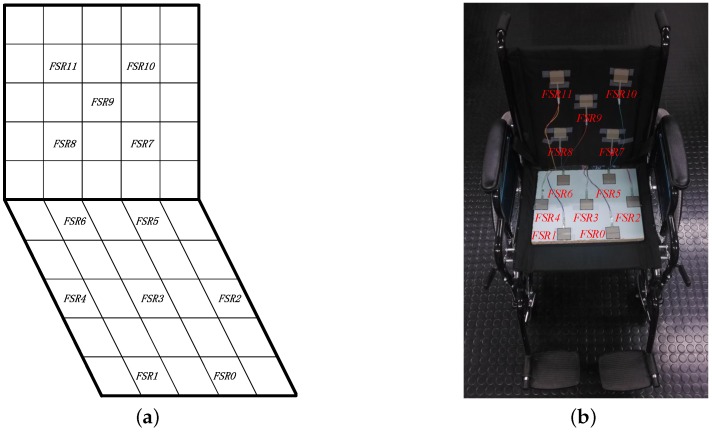
Sensor deployment: (**a**) schematic diagram of the sensor deployment; (**b**) sensors deployed on the real wheelchair.

As previously mentioned, we deployed 12 pressure sensors on the wheelchair (as shown in Figure 3) to evaluate our system. The list of required components along with its market price has been reported in Table 3. The total price of our prototype is $228. In comparison, the commercially available Body Pressure Measurement System (BPMS) using the sensors array developed by TekScan [27] has a sensor matrix of 42×48, and it is sold for over ten thousand US dollars. PoSeat [35] was reported at a price of $460; another marketed smart wheelchair cushion called SENSIMAT [46] is currently sold at $599.

## 4. System Evaluation

Instead of using sensor arrays, we chose to design our cushion with fewer pressure sensors individually connected to the processing unit; this has advantages in terms of economic cost and power consumption.

In Section 2.2, we provide an overview of different kinds of pressure sensor deployment methods. Most of the works listed in Table 2 did not systematically analyzed (or at least described) the deployment methods. In this study, we adopt two systematic principles to determine the sensor deployment; one uses empirical analysis to find the most significant sensors and the other tries to take into account all of the analyzed literature sensor deployment methods.

Here, we adopt a sensor deployment configuration as shown in Figure 3. The deployment is suitable for the body mass distribution. Actually, using the pressure sensors on the seat is enough to detect most of the sitting postures; however, we decided to apply pressure sensors on the backrest too, as a supplementary tool to more comprehensively monitor users’ postures.

Assuming a conventional size for the seat and backrest of 40 cm × 40 cm, we split the seat and backrest into 5 × 5 square zones. We performed a preliminary empirical analysis to exclude the least significant pressure points and found that the most effective initial deployment configuration to detect pressure changes due to different postures is the one depicted in Figure 3. It is worth noting that FSR2 and FSR4 have been deployed to the left and right sides of the seat to better deal with individual physiognomy differences.

In our previous studies [44], we used two pressure sensors on the seat and one pressure sensor on the backrest. However, we noticed that if the user does not sit perfectly at the center of seat, some posture transitions are not correctly detected. Therefore, with the aim of finding a better deployment to cope with different sitting positions, the smart cushion we designed in this work can record up to 12 pressure points simultaneously (the limitation is due to the number of analog inputs of the Arduino DUE platform). All of the FSR sensors (marked as FSR0,…,FSR11) were sampled at a frequency of 2 Hz (i.e., sampling rate of two samples per second) because it is enough to capture posture transitions and has the property of low energy consumption [44].

**Figure 4:**
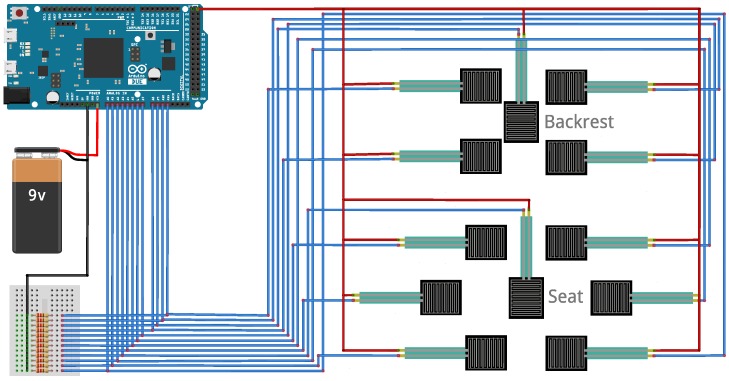
Electrical circuitry of the pressure sensor system.

The electrical circuitry highlighting the connections of the FSR sensors to the Arduino platform is shown in Figure 4.

### 4.1. Posture Definition and Data Collection

We carried out experimental tests in our laboratory involving 12 participants (seven males and five females) with ages in the range of [22, 36] and BMI in the range of [16, 34]. The BMI, whose distribution in our experiment sample is shown in Table 4, is an important parameter to consider in order to realize a user-independent posture recognition system.

To simulate a real-life setting, we asked each participant to perform five different postures that are typical for wheelchair users, as listed in Table 5. When the user sits on the wheelchair, the embedded sensors can measure multiple pressure points due to body weight. The posture data were manually labeled during the experiments; we asked to keep each posture for three minutes; in addition, at the beginning of the experiment and between each posture, we allow 30 s for the participant to adjust the posture in the wheelchair.

The participants were free to choose their favorite seating position and each subject performed the experiment only once. The final dataset, consisting of data collected from all 12 of the test subjects, counts a total of 36,000 different posture recordings (see Table 5).

Pressure sensors signals are marked as {P0,P1,…,P11}. At the time *t*, we obtained the instance vector $Pt=\{Pt_{0},Pt_{1},\ldots ,Pt_{11}\}$. Using this instance vector, we will recognize the sitting postures defined before.

### 4.2. Classification

Studies have shown that a lot of different algorithms can be used for the classification of sitting postures with satisfactory accuracy ranging from 90.9% to 99.5% [36,37,40,44]. Since the performance of the classification results is highly dependent on the used data, we compared the five algorithms as referred to before. In order to choose the best classifier for the classification, we analyzed five supervised machine learning algorithms applied for the recognition of sitting postures. Specifically, we compared J48 decision tree, Support Vector Machine, Multilayer Perceptron, Naive Bayes and k-Nearest Neighbors, respectively, briefly described in the following.

*J48* [50] is a specific decision tree implementation of the well known C4.5 algorithm using the concept of information entropy. The classifier model is generated by a training procedure that uses a set of pre-classified samples. Each sample is a *p*-dimensional vector also known as a feature vector. Each node of the tree represents a decision (typically a comparison against a threshold value); at each node, J48 chooses the feature (i.e., an attribute) of the data that most effectively splits its set of samples into distinct subsets according to the normalized information gain (NIG). In particular, the algorithm chooses the feature with the highest NIG to generate the decision node. The main parameters used to tune the classifier generation are the pruning confidence (*C*) and the minimum number of instances per leaf (*M*), as summarized in Table 6.

*Support Vector Machine (SVM)* [51] is a supervised learning model used for both classification and regression analysis with associated learning algorithms that analyze data to recognize patterns. Given a set of training instances, new samples will be classified with one of the defined categories. The standard SVM builds a model that assigns new instances into one of two possible categories, so the SVM is a type of non-probabilistic binary linear classifier. The key parameters of the SVM classifier are reported in Table 6. In our analysis, we used a specific type of SVM model called regularized support vector classification (C-SVC) and we set the C parameter to 1. We used Radial Basis Function (RBF) as the kernel type with its degree set to 3.

*Multilayer perceptron (MLP)* [52] is a feed-forward artificial neural network model that maps sets of input data onto a set of appropriate outputs. An MLP typically involves three or more layers. MLP adopts a supervised learning technique called back propagation for network training. Except for the input nodes, each node is a neuron (or processing element) with a nonlinear activation function. Nodes are connected with weight values. The network output will be decided by node connection, weight values and activation function. The main parameters of the MLP classifier are reported in Table 6. For our purpose, we need 12 attributes and five classes (corresponding to the five sitting positions), so the model is composed of nine hidden layers. Default Momentum Rate and Learning Rate have been selected for the back-propagation algorithm.

*Naive Bayes* [53] is a simple technique for constructing classifiers: models that assign class labels to problem instances, represented as vectors of feature values, where the class labels are drawn from some finite set. It consists of a family of algorithms based on the assumption that the value of a particular feature is independent from the value of any other feature, given the class variable. An advantage of Naive Bayes is that it only requires a small amount of training data to estimate the parameters necessary for classification. Using different parameters, we trained two classifiers, one using default parameters and the other is BayesNet.

*k-Nearest Neighbors algorithm (k-NN)* [54] is a simple supervised learning method method used for classification and regression. In both cases, the input consists of the k closest training examples in the feature space. The parameter *k* plays the role of a capacity control. If k = 1, it means that the object is simply assigned to the class of that single nearest neighbor.

The five classifiers were set up with various configurations resulting in seven classifier configurations; each classifier was evaluated using the same dataset.

### 4.3. Sensor Deployment Optimization Using the Backward Selection Method

Our goal is to investigate the contribution of each individual sensor to the recognition accuracy and determine the best sensor deployment. This study was performed using the backward selection procedure [55]. It starts with fitting a model with all the variables of interest (i.e., all the 12 pressure sensors active). Then, sensors are excluded one at a time from the base configuration; we choose the best configuration, and the least significant variable is dropped. We iterate this procedure by successively re-fitting reduced models and applying the same rule until only one sensor is left.

## 5. Performance Evaluation

In this section, we present and discuss the results obtained from each classifier and choose the best sensor configuration using the backward selection method. To accurately study the performance evaluation, five relevant metrics (Accuracy, Precision, Recall, F-measure, and Model Build Time) have been compared.

### 5.1. Performance Evaluation of Each Classifier

We analyzed the seven classifiers as in Table 6 using WEKA (Waikato Environment for Knowledge Analysis) data mining toolbox [56] with the dataset described in Section 4.1. Data from all of the 12 pressure sensors are therefore taken into account in this phase. In this work, 10-fold cross validation was used to separate the training from the test set. The classifier models were obviously trained using the training set, while, in the test step, the estimated classes were compared to the true classes in order to compute the classification accuracy.

The Accuracy measure is used to evaluate the classifiers performance; specifically, it measures the proportion of correctly classified instances. In the case of binary classification, the formula of accuracy can be expressed as follows:

(1) $$Accuracy=\frac{Tp+Tn}{Tp+Tn+Fp+Fn}$$

where Tn (true negatives) is the correct classifications of negative examples, Tp (true positives) is the correct classifications of positive examples. Fn (false negatives) and Fp (false positives) are, respectively, the positive examples incorrectly classified into the negative classes and the negative examples incorrectly classified into the positive classes.

For evaluating the classifiers, F-measure accuracy (overall accuracy) of the test data has been computed to evaluate recognition performance. F-measure represents the combination of *precision* and *recall*, defined, respectively, as follows:

(2) $$precision=\frac{Tp}{Tp+Fp}$$

(3) $$recall=\frac{Tp}{Tp+Fn}$$

(4) $$F-measure=\frac{2\cdot recall\cdot precision}{recall+precision}$$

Table 7 summaries the results of our comparison. We recorded the same amount of samples for each different posture. The class imbalance problem was not taken into account for these classifiers. However, the main idea of this paper is to determine which classifier is more suitable for our system.

J48 shows better performance than other classifiers. It is a classifier that predicts the posture classes of a new sample based on the threshold feature values. Furthermore, it is lightweight and easy to implement in embedded devices. We will eventually choose a J48 pruned tree for the implementation on the Arduino platform.

The SVM shows the worst performance among the analyzed classifiers; while it is very effective for binary classification, setting its key parameters for multi classification is much harder. In addition, it is computatively demanding and therefore not easily implemented in embedded and mobile devices. The MLP takes a longer time to build the train model than the J48 and its performance results are lower than J48. MLP also requires high processing resources, making it not ideal on embedded and mobile devices. We did not achieve good results with the Naive Bayes either, especially with configuration No. 4 (see Table 6), and it shows the worst performance among the classifiers we took into consideration. kNN achieved slightly worse accuracy than J48. We observed that different k values did not affect the recognition accuracy: our hypothesis is that the clusters identified by our feature set are internally consistent and well separated among each other. kNN requires less time to build the model (essentially the time required to split the dataset to obtain the training set), but it requires high computation load for classification, so it does not fit lightweight requirements imposed by embedded platforms.

### 5.2. Sensor Selection Using the Backward Selection Method

Given the results described in Section 5.1, we choose J48 as our classifier in the following. It is worth mentioning that the accuracy reported in the table refers to a configuration with all 12 pressure sensors. As described in Section 4.3, sensors were excluded one at a time from the base configuration and accuracy of the recognition was evaluated by a 10-fold cross-validation; then, the configuration with highest accuracy in this step became the base configuration. The procedure then iterates until only one sensor is left. The testing has been performed with a decimation factor d=1.

**Figure 5:**
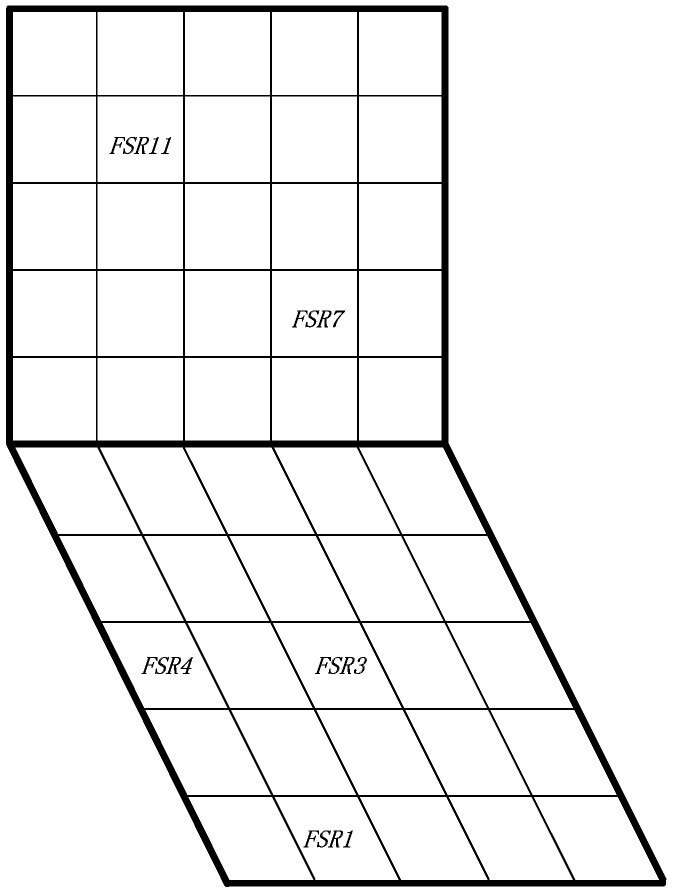
Selected sensors of the best configuration.

Results of the backward selection of various sensor configurations are shown in Table 8. The column represents the number of active sensors in a given configuration. The row represents the ID excluded sensor in a given configuration (let us mention here that once excluded from a configuration, with the backward selection that sensor cannot be re-added to any following iteration). The initial configuration has 12 active sensors achieving an accuracy of 99.48%, as shown in Table 7. At the first iteration of the backward selection procedure, the least significant sensor is FSR10 because the best accuracy that can be achieved with 11 sensors is 99.50% and corresponds to the exclusion of FSR10. Then, the sensors’ configuration during the second iteration is {FSR0,FSR1,FSR2,FSR3,FSR4,FSR5,FSR6,FSR7,FSR8,FSR9,FSR11}; this time, the best configuration can be achieved excluding FSR6, with a maximum possible accuracy of 99.51% (see the bold value in column 10). The procedure continues to iterate excluding, sequentially, FSR8, FSR9, FSR2, FSR5, and FSR0. At this point, the current best configuration has five sensors ({FSR1,FSR3,FSR4,FSR7,FSR11}) with an accuracy of 99.47% as shown in Figure 5. We can conclude that this is the most effective sensor deployment configuration in terms of tradeoff between posture recognition accuracy and number of required sensors (which influences the system complexity and its economic cost). Excluding a further sensor, in fact, would decrease the accuracy below 99%.

### 5.3. Comparison of Recognition Results with Different BMI Values

Using the best sensor configuration determined in Section 5.2, we analyzed the recognition results grouped by the three weight categories classified by BMI (see Section 4.1). In addition, we analyzed the possible advantage of taking the BMI value as one of the features in the dataset.

The results grouped by BMI category obtained without considering the BMI as a feature of the dataset are shown in Table 9 while Table 10 depicts the result of classification accuracy using the dataset with the added BMI feature. Given that we obtained similar results, we can conclude that our sensor deployment can successfully fit different body physiognomies without the need to include user-dependent information (such as his/her BMI) into the classifier model.

### 5.4. Comparison of the Proposed Method with Previous Studies

To compare our current work with the state-of-the-art, we applied the J48 classifier to datasets generated using sensor deployment configurations proposed in other literature works, and we summarized the results in Table 11.

Using the same J48 classifier, we compared the quality of different sensor deployments. It can be clearly seen that our novel proposed deployment method allows to achieve the highest accuracy.

## 6. Conclusions

In this paper, we have proposed an Arduino-based pressure cushion to detect sitting postures of wheelchair users and a sensor selection method to obtain optimal deployment on the wheelchair. We extracted pressure data and performed initial offline analysis using WEKA. Laboratory experiments were run over a diversified sample of subjects (in terms of BMI category) with different sitting habits. Using the obtained dataset, we compared five different machine learning approaches (Decision Tree (J48), Support Vector Machines (SVM), Multilayer Perceptron (MLP), Naive Bayes, and k-Nearest Neighbor (k-NN)) and selected the most effective sensor deployment among the possible configurations given by our smart cushion. We evaluated seven different classifiers to recognize five sitting postures. Results showed that the lightweight J48 algorithm can efficiently and accurately (99.47%) be used for posture recognition of seated users. An advantage of the decision tree-based approach is that trained models are very lightweight and easily implemented on embedded devices. Ongoing efforts are focused on improving current end-user applications based on posture detection. For example, activity level assessment can be achieved based on the analysis of posture transitions. Future research will be devoted to acquiring more data from heterogeneous sensors deployed on the chair, applying multi sensor fusion techniques [57] to better detect the postures, and assessing user activity on a smart wheelchair more accurately.

Finally, we plan to improve our BodyCloud-based Wheelchair System [10,24,58] with the work presented in this paper.

## Figures and Tables

**Table 1:** State-of-the-art on smart cushions based on pressure sensor arrays.

Author	Sensor Array Type	Placement of the Sensors	Detected Postures	Classification Technique/Method	Accuracy
Xu et al. [26]	E-textile	cushion on the seat	Sit up, forward, backward, lean left/right, right foot over left, left over right	Gray scale image	85.9%
Tekscan [27]	E-textile	cushion on the seat and backrest	N/A	Pressure mapping	N/A
Tan et al. [28]	E-textile	cushion on the seat and backrest	N/A	PCA, Grayscale image	96%
Mota et al. [29]	E-textile	cushion on the seat and backrest	Lean forward/back, lean forward right/left, sit upright/on the edge, etc.	Neural Network	87.6%
Meyer et al. [30]	textile pressure sensor	cushion on the seat	Seat upright, lean right, left, forward, back, left leg crossed over right etc.	Naive Bayes	82%
Multu et al. [31]	pressure sensor on the seat and backrest	19 pressure sensors	Left leg crossed, right leg crossed lean left, lean back, lean forward etc.	Logistic Regression	87%
Kamiya et al. [32]	8×8 sensor array	cushion on the seat	Normal, lean forward, lean backward, lean right, right leg crossed, lean right with right leg crossed etc.	SVM	98.9%
Xu et al. [33]	Seat 6×8, backrest 2×8	Cushion on the seat and backrest	Lean left front, lean front, lean right front, lean left, seat upright, lean right etc.	Binary pressure distribution, Naive Bayes	82.3%
Fard et al. [34]	8×8 sensor array	cushion on the seat	Sitting straight with bent keens, crossed legs left to right and right to left, stretched legs	pressure mapping technology	N/A

**Table 2:** Smart cushions based on fewer individual pressure sensors.

Author	Number of Sensors	Placement of the Sensors	Postures Recognized	Classification Techniques	Accuracy
Hu et al. [35]	6	2 on the seat and 4 on the backrest	Sit straight, lean left, lean right, lean back	SVM	N/A
Benocci et al. [36]	5	4 on the seat and 1 on the backrest	Normal posture, right side, left side, right/left/both legs extend forward	kNN	92.7%
Bao et al. [37]	5	5 on the seat	Normal sitting, forward, backward, lean left, lean right, swing, shake	Density-based cluster	94.2%
Diego et al. [38]	4	4 on the seat	N/A	Threshold-based	N/A
Min et al. [39]	6	4 on the seat and 2 on the backrest	Crossing left leg, crossing right leg, forward buttocks, bending down the upper body, correct posture	Decision Tree	N/A
Zemp et al. [40]	16	10 on the seat, 2 on the armrests and 4 on the backrest	Upright, reclined, forward inclined, laterally right/left, crossed legs, left over right/ right over left	SVM, Multinomial Regression, Boosting, Neural Networks and Random Forest	90.9%
Barba et al. [41]	16	8 on the seat and 8 on the backrest	Standard, lying, forward, normal position, sitting on the edge, legs crossed, sitting on one/two foot etc.	N/A	N/A
Fu et al. [42]	8	4 on the seat and 4 on the backrest	N/A	Decision Tree	N/A
Kumar et al. [43]	4	4 on the backrest	N/A	Extremely Randomized Trees	86%
Ma et al. [44]	3	2 on the seat and 1 on the backrest	Upright sitting, lean left, right, forward, backward	Decision Tree	99.5%
Darma [45]	6	6 on the seat	N/A	N/A	N/A
Sensimat [46]	6	6 on the seat	N/A	N/A	N/A

**Table 3:** List of the components required by the prototype system.

Part Name	Description	Price (USD)
Arduino DUE board	Data Processing Unit	30
FSR 406 pressure sensor	12 pressure sensors applied to the seat and backrest	180
Bluetooth shield (HC-06)	Bluetooth module to connect the cushion to mobile devices	8
Seat Cover	A seat cushion	10
Total		228

**Table 4:** Body Mass Index (BMI) distribution of the subjects participating in the experiments.

Description	Underweight	Normal	Overweight and Obese
BMI	<18.5	[18.5, 25)	⩾25
Number of subjects	4	4	4

**Table 5:** Wheelchair user postures of interest.

Posture	Description	Samples of Posture
Proper Sitting (PS)	User seated correctly on the wheelchair	7200
Lean Left (LL)	User seated leaning to the left	7200
Lean Right (LR)	User seated leaning to the right	7200
Lean Forward (LF)	User seated leaning forward	7200
Lean Backward (LB)	User seated leaning backward	7200

**Table 6:** Tested machine learning algorithms.

No.	Classifier	Parameters
1	J48	C = 0.25, M = 2
2	SVM	SVM Type: C-SVC, Kernel Type: RBF, C = 1, Degree = 3
3	MLP	9 hidden layer neurons
4	Naive Bayes	default
5	Naive Bayes	BayesNet
6	kNN	k = 1
7	kNN	k = 5

**Table 7:** Summary of classifiers’ performance.

No.	Classifier	Accuracy	Precision	Recall	F-Measure	Model Build Time (s)
1	J48	**99.48%**	0.995	0.995	0.995	1.98
2	SVM	79.08%	0.880	0.736	0.760	320.34
3	MLP	95.5%	0.926	0.926	0.926	265.46
4	Naive Bayes	49.09%	0.585	0.491	0.427	0.24
5	BayesNet	94.06%	0.945	0.941	0.941	0.93
6	kNN (k = 1)	98.53%	0.995	0.995	0.995	0.04
7	kNN (k = 5)	98.52%	0.995	0.995	0.995	0.08

**Table 8:** Backward selection of the best sensor configuration. Bold font highlights the best accuracy for each number of active sensors.

Number of Active Sensors												
		11	10	9	8	7	6	5	4	3	2	1
**Sensor ID**	0	99.48%	99.49%	99.50%	99.49%	99.50%	99.49%	**99.47%**				
	1	97.06%	97.05%	97.11%	97.11%	97.11%	97.13%	96.51%	92.32%	90.54%	81.23%	**63.98%**
	2	99.49%	99.49%	99.49%	99.50%	**99.51%**						
	3	99.46%	99.50%	99.50%	99.50%	99.50%	99.42%	99.41%	**98.99%**			
	4	99.46%	99.49%	99.50%	99.50%	99.50%	99.46%	99.44%	98.84%	94.36%	87.93%	48.72%
	5	99.48%	99.49%	99.49%	99.49%	99.49%	**99.50%**					
	6	99.49%	**99.51%**									
	7	99.48%	99.49%	99.49%	99.49%	99.49%	99.44%	99.42%	95.80%	92.28%	**89.51%**	
	8	99.48%	99.50%	**99.51%**								
	9	99.49%	99.50%	**99.51%**	**99.51%**							
	10	**99.50%**										
	11	99.48%	99.30%	99.32%	99.33%	99.27%	98.98%	98.98%	98.90%	**98.01%**		
**LSS** ${}^{1}$		FSR10	FSR6	FSR8	FSR9	FSR2	FSR5	FSR0	FSR3	FSR11	FSR7	FSR1

${}^{1}$ Least Significant Sensor.

**Table 9:** Results with different BMI values without considering the BMI feature.

	Accuracy	Precision	Recall	F-Measure
Underweight	99.92%	0.999	0.999	0.999
Normal	98.67%	0.987	0.987	0.987
Overweight and Obese	99.82%	0.998	0.998	0.998
All	99.47%	0.995	0.995	0.995

**Table 10:** Results with different BMI values using the BMI feature in the J48 tree.

	Accuracy	Precision	Recall	F-Measure
Underweight	99.93%	0.999	0.999	0.999
Normal	98.67%	0.987	0.987	0.987
Overweight and Obese	99.83%	0.998	0.998	0.998
All	99.50%	0.995	0.995	0.995

**Table 11:** Recognition results of different sensor deployment.

Author	Sensor Deployment	Accuracy
Hu et al. [35]	6 FSRs (0,1,7,8,10,11)	98.70%
Benocci et al. [36]	5 FSRs (0,1,5,6,9)	97.58%
Bao et al. [37]	5 FSRs (0,1,2,3,4)	99.16%
Diego et al. [38]	4 FSRs (0,1,5,6)	97.11%
Min et al. [39]	4 FSRs (0,1,5,6,10,11)	98.5%
Ma et al. [44]	3 FSRs (2,4,9)	87.25%
Darma [45], Sensimat [46]	6 FSRs (0,1,2,4,5,6)	99.14%
Novel proposed method	5 FSRs (1,3,4,7,11)	**99.47%**
